# Establishing a Dedicated Lung Cancer Biobank at the University Center Hospital of Nice (France). Why and How?

**DOI:** 10.3390/cancers10070220

**Published:** 2018-06-29

**Authors:** Kevin Washetine, Simon Heeke, Christelle Bonnetaud, Mehdi Kara-Borni, Marius Ilié, Sandra Lassalle, Catherine Butori, Elodie Long-Mira, Charles Hugo Marquette, Charlotte Cohen, Jérôme Mouroux, Eric Selva, Virginie Tanga, Coraline Bence, Jean-Marc Félix, Loic Gazoppi, Taycir Skhiri, Emmanuelle Gormally, Pascal Boucher, Bruno Clément, Georges Dagher, Véronique Hofman, Paul Hofman

**Affiliations:** 1 Hospital-Integrated Biobank (BB-0033-00025), Université Côte d’Azur, CHU de Nice, 06001 Nice CEDEX 1, France; washetine.k@chu-nice.fr (K.W.); bonnetaud.c@chu-nice.fr (C.B.); mehdi.kara-borni@etu.unice.fr (M.K.-B.); ilie.m@chu-nice.fr (M.I.); selva.e@chu-nice.fr (E.S.); tanga.v@chu-nice.fr (V.T.); felix.jm@chu-nice.fr (J.-M.F.); gazoppi.l@chu-nice.fr (L.G.); hofman.v@chu-nice.fr (V.H.); 2 Laboratory of Clinical and Experimental Pathology, Université Côte d’Azur, CHU de Nice, University Hospital Federation OncoAge, 06001 Nice CEDEX 1, France; lassalle.s@chu-nice.fr (S.L.); butori.c@chu-nice.fr (C.B.); long-mira.e@chu-nice.fr (E.L.); bence.c@chu-nice.fr (C.B.); 3 Team 4, Institute of Research on Cancer and Aging of Nice (IRCAN), Inserm U1081, CNRS UMR7284, Université Côte d’Azur, CHU de Nice, 06107 Nice CEDEX 2, France; heeke.s@chu-nice.fr (S.H.); marquette.c@chu-nice.fr (C.H.M.); mouroux.j@chu-nice.fr (J.M.); 4 FHU OncoAge, University of Nice Sophia Antipolis, 06001 Nice CEDEX 1, France; cohen.c@chu-nice.fr (C.C.); skhiri.t@chu-nice.fr (T.S.); 5 Department of Pulmonary Medicine and Oncology, Université Côte d’Azur, CHU de Nice, University Hospital Federation OncoAge, 06001 Nice CEDEX 1, France; 6 Department of Thoracic Surgery, Université Côte d’Azur, CHU de Nice, University Hospital Federation OncoAge, 06001 Nice CEDEX 1, France; 7 EPHE, Catholic University of Lyon, 69002 Lyon, France; egormally@univ-catholyon.fr; 8 French National Cancer Institut, 92513 Boulogne Billancourt CEDEX, France; pboucher@institutcancer.fr; 9 INSERM, INRA, University of Rennes, NuMeCan, CRB Santé, CHU Rennes, 35042 Rennes, France; bruno.clement@inserm.fr; 10 INSERM, 75654 Paris, France; georges.dagher@inserm.fr

**Keywords:** lung tumor biobank, efficiency, sustainability, quality, indicators

## Abstract

Lung cancer is the major cause of death from cancer in the world and its incidence is increasing in women. Despite the progress made in developing immunotherapies and therapies targeting genomic alterations, improvement in the survival rate of advanced stages or metastatic patients remains low. Thus, urgent development of effective therapeutic molecules is needed. The discovery of novel therapeutic targets and their validation requires high quality biological material and associated clinical data. With this aim, we established a biobank dedicated to lung cancers. We describe here our strategy and the indicators used and, through an overall assessment, present the strengths, weaknesses, opportunities and associated risks of this biobank.

## 1. Introduction

Several reasons explain the constant increase in the number of research projects in thoracic oncology. Lung cancer is the major cause of death from cancer in the world and its incidence is increasing in the female population [1,2,3]. For advanced stages or metastatic patients, prognosis of this cancer is always poor and no cure allowing a complete recovery is available, despite some recently developed therapies. Some patients with early stage lung cancer relapse after complete surgery due to aggressive tumors for which the biology is still poorly understood. A number of molecular therapeutics have been recently developed. However, only a limited number of therapeutic targets of the many genomic alterations that were identified with high throughput sequencing analysis are available [4,5,6]. The emergence of immunotherapies and of combination therapeutics will require the use of new predictive biomarkers, the evaluation of which is no doubt complex [7,8]. No biological test used in the clinic is able to screen or diagnose the occurrence of early lung cancers. Finally, interest in the analysis of risk factors other than tobacco or of genetic predisposition along with investigation into mechanisms of carcinogenesis in the domain of thoracic oncology is dramatically increasing [9,10].

Fundamental research studies in the area of lung oncology are performed in vitro with cell lines and/or in vivo with animal models. The discovery of novel mechanisms of carcinogenesis will lead to the development of tests for new therapeutic molecules and will define associated predictive biomarkers. Access to large numbers of biological resources (tissues and/or biofluids) collected from patients, which are reliably well clinically annotated, is a mandatory step in the validation of predictive and prognostic biomarkers [11,12]. To define reliable, sensitive and specific biomarkers, a high number of patients must be sampled. However, the heterogeneity of clinical practices and centers for care makes the management and homogenization of the pre-analytical steps difficult [13]. The variability of the quality of the samples obtained from center to center can lead to inconsistent results that are often not reproducible, and for which the economic impact is substantial [14]. In this context, the need for high quality biological resources associated with more and more complex clinical data is constantly increasing [15,16]. Consequently, biobanks must face the challenge to satisfy the requests by scientists and, as the objectives of research projects progress permanently, adapt their management overtime [15,17].

Our aim was to establish and develop a biobank dedicated to lung cancers (Hospital-Integrated Biobank BB-0033-00025) in order to meet the needs of key stakeholders in thoracic oncology research programs. This article describes the strategy adopted to set up and maintain a lung cancer biobank as well as the monitoring indicators for evaluation of performance and the main results obtained since it started in 2006. An analysis of the strengths, weaknesses, opportunities and risks (SWOT) are presented together with a number of perspectives.

## 2. Results

NFS 96-900 certification of the biobank was obtained in 2010 (Certificate N°2010/36548), and was then renewed in 2013 (Certificate N°2010/36548.2) and in 2016 (Certificate N°2010/36548.3). The different biological resources collected by the biobank came from the Nice hospital laboratory (the clinical and experimental laboratory, Pasteur Hospital) that was accredited in 2013 according to the ISO 15189 norm) [18]. Together these standards provide the partners of the biobank with the assurance of the quality of the samples provided [19].

### 2.1. Quantitative Data

**Figure 1:**
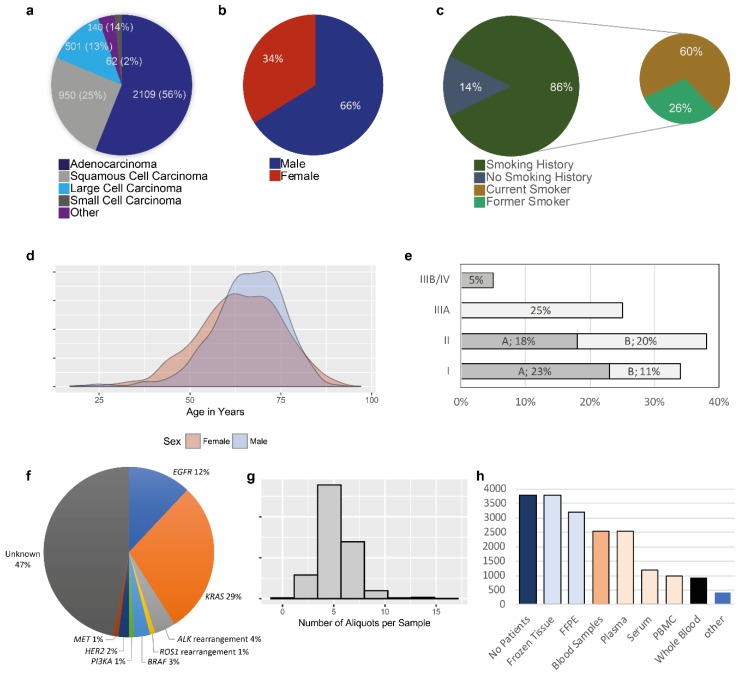
Quantitative data. (**a**) Percentage and total sample number according to each Lung Cancer Histology. (**b**) distribution by sex for all samples stored in the Biobank. (**c**) Smoking history. (**d**) Density plot of the distribution of the age of all patients by sex. (**e**) Percentage of different clinical stagings. Stage A (“A”) and Stage B (“B”) is given individually. (**f**) Main genomic alterations detected in the lung adenocarcinoma patients. (**g**) Histogram demonstrating the number of aliquots for each tumor sample. (**h**) Number of samples stored in the Biobank by sample type.

The main data are shown in Figure 1. A total of 3798 patients who underwent surgery in the department of thoracic surgery of the Nice University Hospital between October 2006 and December 2017 signed informed consent for sampling of biological specimens for research and at least one tissue fragment was obtained from each patient. The latter was essential for a patient to be registered in the biobank database. For the different procedures concerning the collection of the tissue samples, the diagnosis and the selection of these specimens are made by one senior thoracic pathologist (MI, SL, CB, EL, VH, PH) of the Clinical and Experimental Pathology Laboratory (Nice Hospital) during the frozen section activity. The pathology laboratory and the biobank are located in the same laboratory. The surgical rooms are connected to this laboratory with tubes allowing to immediately send the fresh resected samples for frozen section procedures. The main epidemiological data, the type of histology, the molecular characteristics of the lung adenocarcinoma, and the tumor stage based on the pTNM classification are shown in Figure 1a–f. The data concerning frozen tissues (the number of tubes of tumor and non-tumor tissue) are given in Figure 1g. An average of five samples of tumor and five of non-tumor tissue from the same surgical specimen were frozen. FFPE specimens were also collected for 3187/3798 (94%) patients (Figure 1h). Blood samples (10 to 20 mL of whole blood) were obtained from 2541/3798 (67%) patients; the products collected are shown in Figure 1h.

### 2.2. Qualitative Data

**Figure 2:**
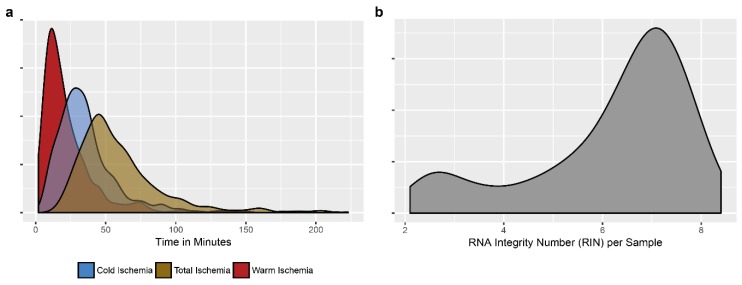
Qualitative data. Some criteria linked to the tissue samples which are recorded are shown; (**a**) warm ischemia time (time from clamping the first pulmonary arteria to the specimen resection) and cold ischemia time (time from getting the resected specimen in operative room until frozen procedure) as well as total ischemia time (as sum of the two) is shown in a density plot; (**b**) Density plot of the RNA integrity number (RIN) in frozen tumor specimens.

The data are shown in Figure 2. The duration of warm ischemia (from clamping the arteries during surgery to resection of the tumor specimen) depended largely on the complexity of the surgical procedures and was consequently very variable (Figure 2a). The surgical specimens were sent from the operating theater to the pathology laboratory via pneumatic tube transport. The cold ischemia time was generally constant (on average 32 min). The percentage of tumor cells was variable (from 5 to 95%). The average RNA integrity number for each selected frozen sample was 6.7 (between 2.1 and 8.4) (Figure 2b). Blood samples were also sent via pneumatic transport to the laboratory. Blood was sampled before starting surgery and the time for transfer tubes from the operating room to the laboratory was no more than 10 min following venipuncture. The handling of the blood samples was done immediately (centrifugation, preparation of PBMC, aliquoting and freezing at -80°C for plasma and sera and in liquid nitrogen for PBMC in DMSO).

### 2.3. Data Concerning the Activity

**Figure 3:**
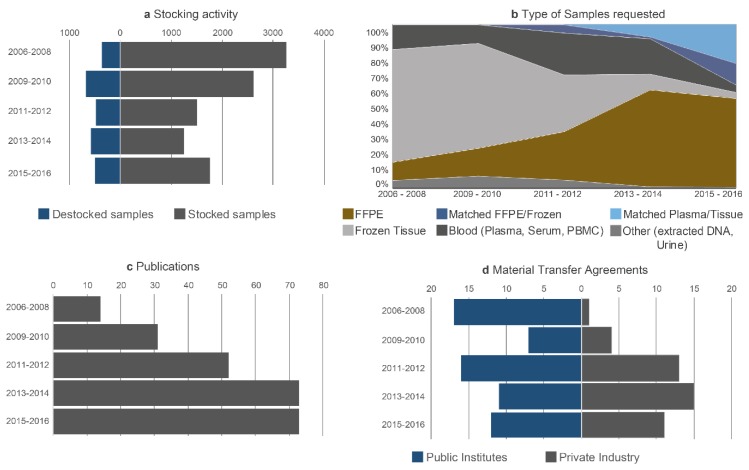
Activity of the Biobank. (**a**) Destocking and stocking of samples given over the mentioned time frames. (**b**) Different types of samples were requested during the years as mentioned in the legend. (**c**) Number of Publications mentioning the Biobank. (**d**) Number of Material Transfer Agreements with public institutions as well as private companies.

The data are shown in Figure 3. The overall number of samples used for research projects compared to the number of samples collected increased progressively as of 2006 and so the ratio of storage/release of samples decreased progressively but stabilized as of 2013 (Figure 3a). Over time, the type of request changed with a decrease in the release of frozen tissue samples and an increase in the number of requests for FFPE samples and for plasma. More particularly, since liquid biopsies began to be part of the clinical routine practice for most of the physicians taking care of lung cancer patients, we observed a strong increase of requests for blood samples and of research projects aiming to look for some predictive biomarkers through this noninvasive test [20,21,22,23]. Requests for several types of samples from the same patient (blood, fixed and/or frozen tissue, blood and fixed tissue) also increased (Figure 3b). The number of publications mentioning the biobank as the source of the biological samples used for research increased progressively over the years (Figure 3c). The studies concerning these publications were performed in partnership with scientists from the academic and industrial sectors [24,25,26,27,28,29,30,31,32,33,34,35,36,37,38,39,40,41,42,43,44,45,46]. The number of contracts concerning release of samples also increased and then stabilized in 2012. These contracts were negotiated together with the research groups or the centers for academic research in 59% of cases and with industrial partners in 41% of cases (Figure 3d). The biobank established expert networks and national and international consortia involved in thoracic oncology (www.oncoage.org) [47,48,49,50,51].

### 2.4. Diffusion of Information and Communication

**Figure 4:**
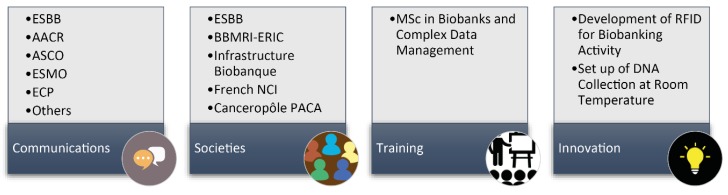
Diffusion of information. Some examples of different action concerning the dissemination concerning the BB-0033-00025 are demonstrated. Examples of congresses where the Nice Biobank team regularly communicates at the international level; membership to different societies; education and training activities at the University Côte d’Azur, Nice, France; Innovative and development projects. Legend: ESBB—European, Middle Eastern and African Society for Biopreservation and Biobanking; AACR—American Association for Cancer Research; ASCO—American Society of Clinical Oncology; ESMO—European Society of Medical Oncology; ECP—European Cancer Prevention; BBMRI-ERIC—Biobanking and Biomolecular Resources Research Infrastructure—European Research Infrastructure Consortium.

To diffuse information concerning the activity and function of the biobank, national and international communications were carried out regularly each year (Figure 4). The visibility of the biobank was also increased through association with different national and international societies involved in biobanking (club 3CR, ESBB, Infrastructure Biobanque France, BBMRI). A master’s degree entitled «biobank and complex data management» was set up by the Université Côte d’Azur (Nice, France) to teach students the profession of biobanking. The teaching was initially developed in partnership with the ESTBB of Lyon [52], and since 2016 a new course was set up in Nice (www.http://univ-cotedazur.fr/archives/english/uca-education/diplomas/biobanks-complex-data). In partnership with several companies including IBM (Armonk, NY, USA) and ST Microelectronic (Geneva, Switzerland), the Nice Biobank developed some innovative technologies. This has led to the development of radio frequency identification (RFID) microchips for cryotubes, which can optimize the traceability of the sample and provide information concerning the patient and the sample [53]. Finally, in collaboration with Imagene (Evry, France) DNA of collections of frozen lung tissue was extracted, which was subsequently lyophilized and encapsulated at room temperature. This allowed us to duplicate these collections, to set up mirror collections and to secure them at several sites.

### 2.5. Economic Model

**Figure 5:**
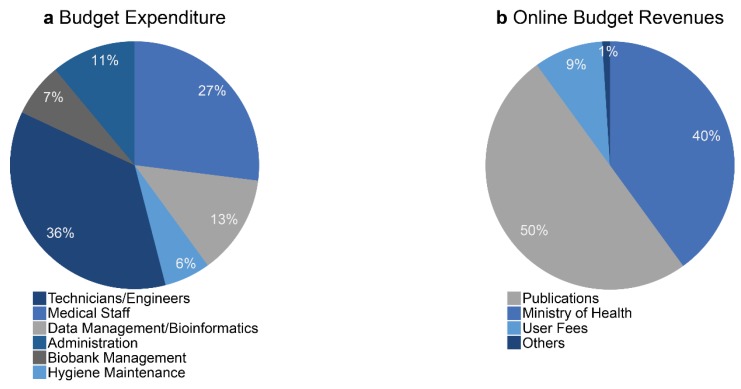
Economic model of the biobank BB-0033-00025. (**a**) Main sources of spending. (**b**) Main sources of income.

The financing of biobanks in France is based in part on an annual renewable budget, which is modified as a function of the activity reported to the «Direction Générale de l’Offre des Soins» (DGOS) in the French Ministry of Health. The activity is evaluated by the DGOS based on: (i) the number of samples collected and released and (ii) the existence of certification (according to the ISO 9001 or NF S96-900 standards). Moreover, a large part of revenue comes from the French Ministry of Health through the budget associated with the MERRI (“missions d’enseignement, de recherche, de reference et d’innovation”). The MERRI include valorisation of the research activity made in the hospitals in France, in particular the number and the value of publications [54]. Additional budget revenue comes from different contracts signed with academic and industrial partnerships asking for samples for research projects (the user fees). Finally, a small part of the revenues comes from budget associated with biorepository activity requested by some partners to store their collection. This budget must at least ensure the salaries of the technical staff of the biobank (Figure 5). The Nice Hospital Center provided secure premises without requesting financial compensation to the biobank. The budget dedicated to the maintenance or replacement of equipment is assured in part by the budget provided by the DGOS and by financing associated with contracts obtained by the biobank (Figure 5).

### 2.6. Identification of the Populations of Interest and Securisation by Sample Duplication

The duplication and storage of the collections at different sites (so called, mirror collections) must assure ideal maintenance of the collections. Among the 3798 lung cancers, we identified a certain number of duplicates. As mentioned above, we extracted and duplicated DNA samples, and conserved the DNA at room temperature after lyophilization using a process of encapsulation. However, since it was difficult to duplicate the whole collection of lung cancer, five major types of samples were duplicated: (i) samples from non-smoker patients, (ii) samples with a sarcomatoid carcinoma diagnosis, (iii) samples with a small cell carcinoma diagnosis, (iv) samples associated with early stage carcinomas of small size (pT1N0M0 stage), and, (v) samples associated with advanced stage or metastatic carcinomas (pTIIIB/IV stage). For the latter two types of tumor we distinguished patients with short survival (<1 year) and those with long survival (>10 years) [55]. Around 15% of the collection of frozen tissues (matched normal and tumoral tissues) was duplicated. We decided to duplicate these samples of interest in a first step, since most of the requests and most of the research projects were associated with these samples.

## 3. Discussion

A certain number of projects associated with the discovery and validation of several biomarkers have emerged after the set up in 2006 and subsequent development of the biobank. These projects were performed together with partners from both the academic and industrial sectors. A modification to the strategy of collection of samples with principally collection of frozen tissue from the same patient; fixed tissue and blood has allowed a number of specific requests to be fulfilled. To perform projects within a scientific collaborative context, we proposed the expertise of the biobank. The implication in the functioning of the biobank of six senior clinical and molecular pathologists (MI, EL, SL, CB, VH, PH) has been very beneficial for the working of the biobank in lung cancer projects [56]. The increase in the number of projects and in the release of samples associated with an MTA have allowed sustainability and then optimization of the functioning of the biobank. Certification of the biobank located in a hospital medical laboratory accredited according to the ISO 15189 norm, has certainly been an important factor for the recognition by academic and industrial partners with whom collaborative scientific programs were rapidly initiated. The biobank works closely with research groups studying lung cancers. This has led to publications and consequently an increase in the visibility of the biobank with respect to scientists wishing to obtain samples.

**Figure 6:**
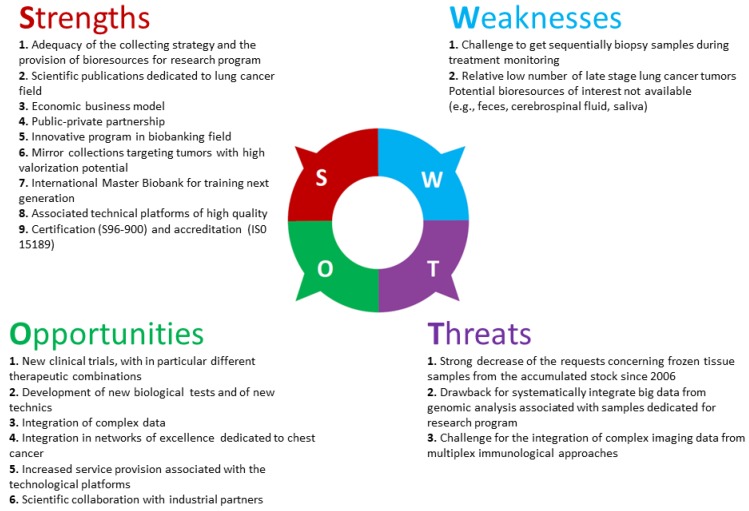
SWOT analysis of the biobank BB-0033-00025.

To better orientate short- and long-term strategies a “SWOT” analysis was performed. The main results of this analysis are given in Figure 6. The major Strengths included: (i) a good balance between the policy of the collection and the availability of samples for research projects, (ii) publication of studies into lung cancer field that were made possible through the analysis of samples obtained from the biobank, (iii) an economic model authorizing sustainability of the activities but also allowing investments, which was made possible partly as a result of public-private partnerships which took the interests of the respective parties into account [57], (iv) the development of innovative projects at the biobank, (v) the duplication of collections of strong interest to ensure their safety, (vi) the creation of an international master of sciences (MSc) dedicated to the management of biobanks and complex data and finally, (vii) the integration of technical platforms allowing service provision but also the development of research projects. The Opportunities included: (i) substantial development of clinical trials using novel therapeutics (targeted therapies and immunotherapies); the validation of biomarkers must use robust tests employing high quality blood and/or tissue samples, (ii) integration of validated sensitive and specific novel technologies using biological samples, (iii) the need to collect increasing amounts of increasingly complex clinical and biological data, in particular genome sequencing data, as well as the participation of several staff members (clinicians, pathologists and biologists, but also mathematicians, information scientists, lawyers and scientists related to social and human sciences) who optimize the collection and use of the biological resources and, (iv) participation in thematic networks of excellence to bring the competence needed to reply to requests of ambitious calls for proposals together; which is a particularly important objective to be developed and maintained by the biobank [58]. The Weaknesses that emerged from our study included: (i) the difficulty to collect sequential clinical and biological data associated to the progress of the patient after surgery, particularly aside from the blood samples obtained during regular follow-up, it is difficult to obtain these tissue samples. Indeed, if obtained these samples are generally dedicated to decisions concerning therapeutic strategies and/or clinical trials, (ii) the low number of tissue samples from advanced or metastatic phases (stage IIIB/IV) since systematic collection of biopsies (bronchial or transthoracic samples) at the biobank is difficult to implement, and, (iii) the current absence of certain biological sources of interest for future research projects (feces, cerebrospinal fluid and saliva). The identified Threats included: (i) the rapid decrease in requests for frozen tissue samples when a high number of samples are already stored, which would not decrease due to the absence of the release of samples, (ii) the possibility of failing to integrate genomic data in a prospective and systematic way, (iii) the impossibility of connecting complex information obtained from newly developed morphological analyses (immunohistochemistry associated with multiplexing analysis), from genetic analyses and the increasingly voluminous clinical data.

After performing the SWOT analysis, a number of measures were adopted by the biobank to strengthen the work and activities (Table 1).

Future perspectives on the biobank BB-003-00025 are to integrate many information and complex data associated with the collected biological samples. In this context, the biobank database should be able to record different data obtained from research projects made with these biological samples in the future (such as whole genome sequencing and information from multiplex immunohistochemistry). One perspective would be to collect not only tissues from surgically resected specimen, but also biopsies obtained during endoscopy and transthoracic biopsies as well as specimen collected during fine needle aspiration of thoracic tumors. Finally, the collection of sequential biopsies obtained during treatment monitoring (at baseline, after surgery or after the first line of treatment, before and during the lung cancer relapse) will improve the development of projects aiming to look for new prognosis and predictive biomarkers.

## 4. Materials and Methods

### 4.1. Global Strategy of the Nice Hospital-Integrated Lung Tumor Biobank/BB-0033-00025

The biobank dedicated to biological samples collected from patients with lung cancer who were hospitalized at the Nice University Hospital (Pasteur Hospital, Nice, France) was set up and started to operate in October 2006. Keeping in mind that this domain is highly competitive, we adopted a strategy according to the different points cited below. The reasons for choosing to establish a biobank dedicated to thoracic oncology at the Nice University Hospital included: (i) the motivation of a multidisciplinary team composed of thoracic surgeons, lung oncologists, thoracic pathologists, biologists, statisticians, scientists working in lung cancer field and biobankers, (ii) the opportunity to rapidly integrate academic and industrial; local, national and international, research programs concerning lung cancer, (iii) the capacity to prospectively collect clinical data associated to biological information in a database, including the follow up and the clinical outcome of patients. The choice of lung cancer pathology was also guided by the number of patients hospitalized and treated annually for lung cancer at the Nice University Hospital (around 500 patients). From the start, it was considered essential to adopt internationally recognized standards of good practice for the function of the biobank [59]. We chose to diversify the types of biological resources collected from patients, including tissues frozen or fixed in formalin and embedded in paraffin (FFPE) (tumor or non-tumor lung tissue), whole blood and different blood products [plasma, serum, peripheral blood mononuclear cell (PBMC)]. The strategy of a diversified collection was subsequently adapted in a prospective way to other collections (urine, pleural effusion fluid) in response to the requests made by research scientists [17,60,61,62].

Selection of some populations of interest having a lung cancer was made in association with the scientific committee of the biobank. To ensure safety and security of these collections of strong value, they were duplicated and stored at several sites (creation of mirror collections).

A policy of pricing of the samples provided to scientists and of the formulation of contracts and material transfer agreements (MTA) was elaborated in association with the research and innovation department of the Nice University Hospital. The evaluation of these costs and their application followed the recommendations formulated at a national and international level by taking the amount of investment by the biobank and the level of the scientific collaboration into consideration [63,64]. The contracts signed by the scientists required them to cite the biobank in their publications, at different levels depending on the implication of the members of biobank (co-author, citation in the material and methods section or in the acknowledgments section). Initially the terminology referring to the biobank in these studies was rather variable, but to increase the recognition at the external level, the identity «BB-0033-00025» was consequently adopted, complying with international recommendations [65,66]. The systematic collection of samples followed a precise protocol that included signed consent from patients in the presence of the clinician after reading an informative note explaining the aims of the collection of the samples [67]. An application for certification according to procedures according to the S96-900 norm used in France was initiated in 2009 [68].

### 4.2. Surrogate Indicators

The indicators we used for monitoring were defined by expert working groups [69,70], however they have been simplified in this article. Here, we give the main criteria followed since the creation of the biobank. Over a period of 11 years, we collected information based on the following elements:

(i) Quantitative data. The number of patients who gave signed informed consent for the collection of their biological samples was registered each year. Frozen tumor tissue samples were initially registered in the clinical-biological database. Associated clinical data to the biological samples collection included smoking and professional exposure (s) history (obtained by means of a questionnaire), patient and family medical history (including history of cancer), follow up after surgery [tumor recurrence, metastatic site (s), progression free survival, treatment, overall survival]. The aim was to associated the frozen tumor tissues with some other biological resources including ideally for each patient frozen non-tumor tissue, fixed tumor and non-tumor tissue included in paraffin, blood samples (whole blood, plasma, serum, PBMC). The fragments of fresh tissue selected by senior thoracic pathologists were placed in cryotubes and weighed before freezing. Depending on the size of the tumor and on the presence of non-tumor tissue several fragments were frozen with a limit of eight fragments for each tumor and non-tumor tissue. Likewise, the number of fixed tissue blocks and of tubes of sampled blood depended on the amount of the available biological resource.
(ii) Qualitative data. The duration of warm and cold ischemia time for the tissue and of the delay between the sampling of the blood and delivery/centrifugation in the laboratory were registered systematically. Quality controls were performed monthly on two randomly selected tissues from cryotubes (one frozen tumor tissue and one frozen non-tumor tissue) by analyzing the RNA integrating number on an Agilent bioanalyzer. A block of fixed tissue (tumor and non-tumor), taken as a mirror sample of the frozen sample, was sectioned and stained with hematoxylin eosin to confirm the tumor and non-tumor character of the corresponding frozen sample and to quantify the percentage of tumor cells and the presence of area of necrosis. These qualitative data reflected the handling of the pre-analytical phase, indicators that are critical to the management of the biobank [71,72,73].
(iii) Data linked to the work. The ratio between the number of samples collected and stored and the number of samples used for research programs (stocking–destocking ratio) was determined annually. The accumulated impact factor of the publications citing the biobank was also noted annually. The number of contracts and MTAs signed each year was monitored, distinguishing between the contracts signed by the academic and industrial partners, both at the national and international levels. Participation in thematic networks, groups of experts and consortium of excellence and in granted research projects was included in this chapter.
(iv) Data associated to the dissemination of the activity of the biobank. This is certainly a challenging criteria to be used for evaluation but several elements were noted: The number of conference presentations, in particular on a topic relative to biobanking, the affiliation of the biobank to international societies of biobanking, training support and teaching related to the activities of biobanks.
(v) Economic data. The most critical indicators to monitor include the revenues and expenses since they finally allow the biobank to adapt its working strategy. Annual evaluation of the budget balance is required to invest, pay staff, maintain the equipment and develop innovative projects.

## 5. Conclusions

The main missions of a biobank focusing on lung cancer include: (i) the possibility to provide high quality and perfectly annotated clinical samples to research scientists to improve knowledge in the pathophysiology and/or to discover and validate specific and sensitive biomarkers, and, (ii) the opportunity to participate in the development of new treatments. The BB-0033-00025 biobank will face a number of challenges in the future. Nowadays, it is necessary to regularly and systematically collect samples (fixed and/or frozen tissues, blood, urine, etc.) before and after treatment, before each treatment modification or during periods of loco-regional tumor progression as well as at each metastatic episode [74,75]. The possibility of developing intravital biobanks provides an opportunity to test novel therapeutics in in vivo models [76]. This will be a major contributor to research different biomarkers of resistance and tumor progression [76]. The automatic acquisition of digital images of the morphology of the different frozen and/or fixed tumor tissues provides an added value to the biobank, permitting easy access to important information such as the histological type, the results of immunohistochemical analyses, the percentage of tumor cells and the areas of necrosis [77].

A biobank dedicated to a defined pathology must also possess expert staff and should participate in the set up and development of research projects. Thus, a biobank must attain recognition through scientific publications. Therefore, in addition to collecting samples and clinical information, performing quality control and ensuring secure storage, a biobank must provide an associated technological platform that permits participation in projects as well as providing a service that assures a durable economic model. These include the platforms for immunohistochemistry and in situ hybridization using multiplexing approaches, genomic targeting (qPCR, digital PCR), next-generation sequencing, tissue microarrays, analysis of circulating tumor cells, etc. [28,61].

One of the fears of the head of the biobank relates to the accumulation of biological resources for which no requests are formulated. This can occur independently of the initial set up of the biobank and the available budget. So, to ensure the function and optimization of the external visibility, it is essential to maintain a dynamic strategy and thinking process concerning the approaches of choice by involving all the members of the biobank.

## Figures and Tables

**Table 1:** Key actions and policy orientations.

Main Bottlenecks and Limitations in Current Functioning of the BB-0033-00025	Proposed Solutions to Improve the Future Development for Research Projects
- Absence of specific collections (feces, saliva, etc.) - Low number of tumor samples from stage III/IV - Absence of tissue samples taken sequentially in treated stage III/IV patients - Considerable quantity accumulation of frozen tissue samples without end-user requests - Possibilities to do easy complex data request (including large sequencing and highplex immunochemistry results)	- To be set up systematically in 2018 in stage III/IV patients only - New procedure allowing the transfer of some FFPE lung biopsies stored in the pathology lab to the biobank * - Getting authorization from local ethic committee to obtain samples during patient monitoring ** - Interruption of frozen procedure for tumor tissues *** - Developing software for complex needs

* FFPE blocks from dead patients will be included in the biobank unless objection of the patient was notified during his lifetime. ** Most of these samples could be obtained during clinical trials (and then stored in the biobank after using and according to a favorable decision from the ethic committee) or for diagnostic reasons. *** This has to be challenged case per case according to some histological types of lung cancer (as an example if small cell lung carcinoma, frozen procedure should continue).

## References

[1] Didkowska J., Wojciechowska U., Mańczuk M., Łobaszewski J. (2016). Lung cancer epidemiology: Contemporary and future challenges worldwide. Ann. Transl. Med..

[2] Siegel R.L., Miller K.D., Jemal A. (2017). Cancer Statistics, 2017. CA Cancer J. Clin..

[3] Torre L.A., Islami F., Siegel R.L., Ward E.M., Jemal A. (2017). Global Cancer in Women: Burden and Trends. Cancer Epidemiol. Biomark. Prev..

[4] Devarakonda S., Masood A., Govindan R. (2017). Next-Generation Sequencing of Lung Cancers: Lessons Learned and Future Directions. Hematol. Oncol. Clin. North Am..

[5] Oberndorfer F., Müllauer L. (2018). Molecular pathology of lung cancer: Current status and perspectives. Curr. Opin. Oncol..

[6] Schallenberg S., Merkelbach-Bruse S., Buettner R. (2017). Lung cancer as a paradigm for precision oncology in solid tumours. Virchows Arch..

[7] Khagi Y., Kurzrock R., Patel S.P. (2017). Next generation predictive biomarkers for immune checkpoint inhibition. Cancer Metastasis Rev..

[8] Mascaux C., Tsao M.-S., Hirsch F.R. (2018). Genomic Testing in Lung Cancer: Past, Present, and Future. J. Natl. Compr. Cancer Netw..

[9] Akhtar N., Bansal J.G. (2017). Risk factors of Lung Cancer in nonsmoker. Curr. Probl. Cancer.

[10] De Matteis S., Heederik D., Burdorf A., Colosio C., Cullinan P., Henneberger P.K., Olsson A., Raynal A., Rooijackers J., Santonen T. (2017). European Respiratory Society Environment and Health Committee Current and new challenges in occupational lung diseases. Eur. Respir. Rev..

[11] Hewitt R.E. (2011). Biobanking: The foundation of personalized medicine. Curr. Opin. Oncol..

[12] Zatloukal K., Hainaut P. (2010). Human tissue biobanks as instruments for drug discovery and development: Impact on personalized medicine. Biomark. Med..

[13] Plebani M. (2010). The detection and prevention of errors in laboratory medicine. Ann. Clin. Biochem..

[14] Freedman L.P., Cockburn I.M., Simcoe T.S. (2015). The Economics of Reproducibility in Preclinical Research. PLoS Biol..

[15] Botti G., Franco R., Cantile M., Ciliberto G., Ascierto P.A. (2012). Tumor biobanks in translational medicine. J. Transl. Med..

[16] Botti G., De Cecio R., Cantile M. (2017). Tumor biobank as fundamental bio-resource for RNA analysis technologies. Minerva Biotecnol..

[17] Braun L., Lesperance M., Mes-Massons A.-M., Tsao M.S., Watson P.H. (2014). Individual Investigator Profiles of Biospecimen Use in Cancer Research. Biopreserv. Biobank..

[18] Washetine K., Long E., Hofman V., Lassalle S., Ilie M., Lespinet V., Bonnetaud C., Bordone O., Gavric-Tanga V., Selva E. (2013). The accreditation of a surgical pathology and somatic genetic laboratory (LPCE, CHU of Nice) according to the ISO 15189 norm: Sharing of experience. Ann. Pathol..

[19] Carter A., Betsou F. (2011). Quality assurance in cancer biobanking. Biopreserv. Biobank..

[20] Hofman P. (2017). Liquid biopsy and therapeutic targets: Present and future issues in thoracic oncology. Cancers.

[21] Hofman P. (2017). Liquid biopsy for early detection of lung cancer. Curr. Opin. Oncol..

[22] Ilie M., Hofman V., Long E., Bordone O., Selva E., Washetine K., Marquette C.H., Hofman P. (2014). Current challenges for detection of circulating tumor cells and cell-free circulating nucleic acids, and their characterization in non-small cell lung carcinoma patients. What is the best blood substrate for personalized medicine?. Ann. Transl. Med..

[23] Rolfo C., Mack P.C., Scagliotti G.V., Baas P., Barlesi F., Bivona T.G., Herbst R.S., Mok T.S., Peled N., Pirker R., Raez L.E. (2018). IASLC Statement Paper: Liquid Biopsy for Advanced Non-Small Cell Lung Cancer (NSCLC). J. Thorac. Oncol..

[24] Brahimi-Horn M.C., Ben-Hail D., Ilie M., Gounon P., Rouleau M., Hofman V., Doyen J., Mari B., Shoshan-Barmatz V., Hofman P. (2012). Expression of a truncated active form of VDAC1 in lung cancer associates with hypoxic cell survival and correlates with progression to chemotherapy resistance. Cancer Res..

[25] Fouret R., Laffaire J., Hofman P., Beau-Faller M., Mazieres J., Validire P., Girard P., Camilleri-Broet S., Vaylet F., Leroy-Ladurie F. (2012). A Comparative and Integrative Approach Identifies ATPase Family, AAA Domain Containing 2 as a Likely Driver of Cell Proliferation in Lung Adenocarcinoma. Clin. Cancer Res..

[26] Italiano A., Cortot A.B., Ilie M., Martel-Planche G., Fabas T., Pop D., Mouroux J., Hofman V., Hofman P., Pedeutour F. (2009). EGFR and KRAS status of primary sarcomatoid carcinomas of the lung: Implications for anti-EGFR treatment of a rare lung malignancy. Int. J. Cancer.

[27] Job B., Bernheim A., Beau-Faller M., Camilleri-Broët S., Girard P., Hofman P., Mazières J., Toujani S., Lacroix L., Laffaire J. (2010). LG Investigators Genomic aberrations in lung adenocarcinoma in never smokers. PLoS ONE.

[28] Hofman V., Ilie M.I., Long E., Selva E., Bonnetaud C., Molina T., Vénissac N., Mouroux J., Vielh P., Hofman P. (2011). Detection of circulating tumor cells as a prognostic factor in patients undergoing radical surgery for non-small-cell lung carcinoma: Comparison of the efficacy of the CellSearch Assay^™^ and the isolation by size of epithelial tumor cell method. Int. J. Cancer.

[29] Hofman P., Ilie M., Hofman V., Roux S., Valent A., Bernheim A., Alifano M., Leroy-Ladurie F., Vaylet F., Rouquette I. (2012). Immunohistochemistry to identify EGFR mutations or ALK rearrangements in patients with lung adenocarcinoma. Ann. Oncol..

[30] Ilie M.I., Hofman V., Bonnetaud C., Havet K., Lespinet-Fabre V., Coëlle C., Gavric-Tanga V., Vénissac N., Mouroux J., Hofman P. (2010). Usefulness of tissue microarrays for assessment of protein expression, gene copy number and mutational status of EGFR in lung adenocarcinoma. Virchows Arch..

[31] Ilie M., Mazure N.M., Hofman V., Ammadi R.E., Ortholan C., Bonnetaud C., Havet K., Venissac N., Mograbi B., Mouroux J. (2010). High levels of carbonic anhydrase IX in tumour tissue and plasma are biomarkers of poor prognostic in patients with non-small cell lung cancer. Br. J. Cancer.

[32] Ilie M., Khambata-Ford S., Copie-Bergman C., Huang L., Juco J., Hofman V., Hofman P. (2017). Use of the 22C3 anti-PD-L1 antibody to determine PD-L1 expression in multiple automated immunohistochemistry platforms. PLoS ONE.

[33] Ilie M., Juco J., Huang L., Hofman V., Khambata-Ford S., Hofman P. (2018). Use of the 22C3 anti-programmed death-ligand 1 antibody to determine programmed death-ligand 1 expression in cytology samples obtained from non-small cell lung cancer patients. Cancer Cytopathol..

[34] Ilie M., Szafer-Glusman E., Hofman V., Long-Mira E., Suttmann R., Darbonne W., Butori C., Lalvée S., Fayada J., Selva E. (2017). Expression of MET in circulating tumor cells correlates with expression in tumor tissue from advanced-stage lung cancer patients. Oncotarget.

[35] Mazières J., Rouquette I., Lepage B., Milia J., Brouchet L., Guibert N., Beau-Faller M., Validire P., Hofman P., Fouret P. (2013). Specificities of Lung Adenocarcinoma in Women Who Have Never Smoked. J. Thorac. Oncol..

[36] Ilie M.I., Hofman V., Ortholan C., Ammadi R. El, Bonnetaud C., Havet K., Venissac N., Mouroux J., Mazure N.M., Pouysségur J., Hofman P. (2011). Overexpression of carbonic anhydrase XII in tissues from resectable non-small cell lung cancers is a biomarker of good prognosis. Int. J. Cancer.

[37] Puisségur M.-P., Mazure N.M., Bertero T., Pradelli L., Grosso S., Robbe-Sermesant K., Maurin T., Lebrigand K., Cardinaud B., Hofman V. (2011). miR-210 is overexpressed in late stages of lung cancer and mediates mitochondrial alterations associated with modulation of HIF-1 activity. Cell Death Differ..

[38] Rakha E., Pajares M.J., Ilie M., Pio R., Echeveste J., Hughes E., Soomro I., Long E., Idoate M.A., Wagner S. (2015). Stratification of resectable lung adenocarcinoma by molecular and pathological risk estimators. Eur. J. Cancer.

[39] Sanfiorenzo C., Ilie M.I., Belaid A., Barlési F., Mouroux J., Marquette C.-H., Brest P., Hofman P. (2013). Two panels of plasma microRNAs as non-invasive biomarkers for prediction of recurrence in resectable NSCLC. PLoS ONE.

[40] Ilie M., Long E., Butori C., Hofman V., Coelle C., Mauro V., Zahaf K., Marquette C.H., Mouroux J., Paterlini-Bréchot P. (2012). ALK-gene rearrangement: A comparative analysis on circulating tumour cells and tumour tissue from patients with lung adenocarcinoma. Ann. Oncol..

[41] Ilie M., Hofman V., Ortholan C., Bonnetaud C., Coëlle C., Mouroux J., Hofman P. (2012). Predictive clinical outcome of the intratumoral CD66b-positive neutrophil-to-CD8-positive T-cell ratio in patients with resectable nonsmall cell lung cancer. Cancer.

[42] Ilie M., Hofman V., Zangari J., Chiche J., Mouroux J., Mazure N.M., Pouysségur J., Brest P., Hofman P. (2013). Response of CAIX and CAXII to in vitro re-oxygenation and clinical significance of the combined expression in NSCLC patients. Lung Cancer.

[43] Ilie M., Long E., Hofman V., Dadone B., Marquette C.H., Mouroux J., Vignaud J.M., Begueret H., Merlio J.P., Capper D. (2013). Diagnostic value of immunohistochemistry for the detection of the BRAFV600E mutation in primary lung adenocarcinoma Caucasian patients. Ann. Oncol..

[44] Ilie M., Nunes M., Blot L., Hofman V., Long-Mira E., Butori C., Selva E., Merino-Trigo A., Vénissac N., Mouroux J. (2015). Setting up a wide panel of patient-derived tumor xenografts of non-small cell lung cancer by improving the preanalytical steps. Cancer Med..

[45] Ilié M., Szafer-Glusman E., Hofman V., Chamorey E., Lalvée S., Selva E., Leroy S., Marquette C.-H., Kowanetz M., Hedge P. (2018). Detection of PD-L1 in circulating tumor cells and white blood cells from patients with advanced non-small-cell lung cancer. Ann. Oncol..

[46] Ilie M., Long E., Hofman V., Selva E., Bonnetaud C., Boyer J., Vénissac N., Sanfiorenzo C., Ferrua B., Marquette C.-H. (2014). Clinical value of circulating endothelial cells and of soluble CD146 levels in patients undergoing surgery for non-small cell lung cancer. Br. J. Cancer.

[47] Chabannon C., Honstettre A., Daufresne L.-M., Martin P.-M., Bonnetaud C., Birtwisle-Peyrottes I., Romain S., Achache K., Mery O., Bordonne O. (2010). Publication of biological samples collections catalogues by tumor banks. Bull. Cancer.

[48] Chalabreysse L., Thomas De Montpreville V., De Muret A., Hofman V., Lantuejoul S., Parrens M., Payan M.-J., Rouquette I., Secq V., Girard N. (2014). Rythmic-pathology: The French national pathology network for thymic epithelial tumours. Ann. Pathol..

[49] Doucet M., Becker K.F., Björkman J., Bonnet J., Clément B., Daidone M.-G., Duyckaerts C., Erb G., Haslacher H., Hofman P. (2017). Quality Matters: 2016 Annual Conference of the National Infrastructures for Biobanking. Biopreserv. Biobank..

[50] Galateau-Sallé F., Gilg Soit Ilg A., Le Stang N., Brochard P., Pairon J.C., Astoul P., Frenay C., Blaizot G., Chamming’s S., Ducamp S. (2014). The French mesothelioma network from 1998 to 2013. Ann. Pathol..

[51] (2011). The Biobank Unit of the Centre Hospitalo-Universitaire (CHU) de Nice (Biobank06). Biopreserv. Biobank..

[52] Gormally E., Hardy I., Caboux E., di Donato J.-H., Hainaut P., Hofman P. (2017). Training the Next Generation of Biobankers: A Two-Year Master’s Course in the Management of Biobanks. Biopreserv. Biobank..

[53] Lagardere C.H.P. French Biobank Secures Specimens. http://www.rfidjournal.com/article/view/8303.

[54] Les missions d’enseignement, de recherche, de référence et d’innovation—MERRI. http://solidarites-sante.gouv.fr/systeme-de-sante-et-medico-social/recherche-et-innovation/l-innovation-et-la-recherche-clinique/article/les-missions-d-enseignement-de-recherche-de-reference-et-d-innovation-merri.

[55] Washetine K., Kara-borni M., Heeke S., Bonnetaud C., Jean-marc F., Ribeyre L., Bence C., Ili M., Bordone O., Pedro M. (2018). Ensuring the Safety and Security of Frozen Lung Cancer Tissue Collections through the Encapsulation of Dried DNA. Cancers.

[56] Bevilacqua G., Bosman F., Dassesse T., Höfler H., Janin A., Langer R., Larsimont D., Morente M.M., Riegman P., Schirmacher P. (2010). The role of the pathologist in tissue banking: European Consensus Expert Group Report. Virchows Arch..

[57] Hofman P., Bréchot C., Zatloukal K., Dagher G., Clément B. (2014). Public–private relationships in biobanking: A still underestimated key component of open innovation. Virchows Arch..

[58] Hewitt R., Hainaut P. (2011). Biobanking in a Fast Moving World: An International Perspective. JNCI Monogr..

[59] Vaught J., Lockhart N.C. (2012). The evolution of biobanking best practices. Clin. Chim. Acta.

[60] Castillo-Pelayo T., Babinszky S., LeBlanc J., Watson P.H. (2015). The Importance of Biobanking in Cancer Research. Biopreserv. Biobank..

[61] Cole A., Cheah S., Dee S., Hughes S., Watson P.H. (2012). Biospecimen Use Correlates with Emerging Techniques in Cancer Research: Impact on Planning Future Biobanks. Biopreserv. Biobank..

[62] Hughes S.E., Barnes R.O., Watson P.H. (2010). Biospecimen use in cancer research over two decades. Biopreserv. Biobank..

[63] Clément B., Yuille M., Zaltoukal K., Wichmann H.-E., Anton G., Parodi B., Kozera L., Bréchot C., Hofman P., Dagher G. (2014). EU-US Expert Group on cost recovery in biobanks Public biobanks: Calculation and recovery of costs. Sci. Transl. Med..

[64] Gonzalez-Sanchez M.B., Lopez-Valeiras E., Morente M.M., Fernández Lago O. (2013). Cost Model for Biobanks. Biopreserv. Biobank..

[65] Cambon-Thomsen A., Thorisson G.A., Mabile L. (2011). BRIF Workshop Group The role of a bioresource research impact factor as an incentive to share human bioresources. Nat. Genet..

[66] Mabile L., Dalgleish R., Thorisson G.A., Deschênes M., Hewitt R., Carpenter J., Bravo E., Filocamo M., Gourraud P.A., Harris J.R. (2013). Quantifying the use of bioresources for promoting their sharing in scientific research. Gigascience.

[67] Hofman V., Bonnetaud C., Gaziello M.C., Ilie M., Lassalle S., Butori C., Lerda N., Selva E., Gavric-Tanga V., Castillo L. (2010). The Nice CHU biobank experience to collect patients’ informed consent for research context (2004–2009). Ann. Pathol..

[68] https://www.boutique.afnor.org/norme/v2/nf-s96-900/qualite-des-centres-de-ressources-biologiques-crb-systeme-de-management-d-un-crb-et-qualite-des-ressources-biologiques/article/747984/fa169771.

[69] Hofman V., Ilie M., Long E., Washetine K., Chabannon C., Figarella-Branger D., Clément B., Mabile L., Cambon-Thomsen A., Boucher P. (2013). Measuring the contribution of tumor biobanks to research in oncology: Surrogate indicators and bibliographic output. Biopreserv. Biobank..

[70] Yong W.H., Dry S.M., Shabihkhani M. (2014). A practical approach to clinical and research biobanking. Methods Mol. Biol..

[71] Ellervik C., Vaught J. (2015). Preanalytical variables affecting the integrity of human biospecimens in biobanking. Clin. Chem..

[72] Hubel A., Spindler R., Skubitz A.P.N. (2014). Storage of human biospecimens: Selection of the optimal storage temperature. Biopreserv. Biobank..

[73] Ma Y., Dai H., Kong X. (2012). Impact of warm ischemia on gene expression analysis in surgically removed biosamples. Anal. Biochem..

[74] Basik M., Aguilar-Mahecha A., Rousseau C., Diaz Z., Tejpar S., Spatz A., Greenwood C.M.T., Batist G. (2013). Biopsies: Next-generation biospecimens for tailoring therapy. Nat. Rev. Clin. Oncol..

[75] Diaz Z., Aguilar-Mahecha A., Paquet E.R., Basik M., Orain M., Camlioglu E., Constantin A., Benlimame N., Bachvarov D., Jannot G. (2013). Next-generation biobanking of metastases to enable multidimensional molecular profiling in personalized medicine. Mod. Pathol..

[76] Luo J., Guo X.-R., Tang X.-J., Sun X.-Y., Yang Z.-S., Zhang Y., Dai L.-J., Warnock G.L. (2014). Intravital biobank and personalized cancer therapy: The correlation with omics. Int. J. Cancer.

[77] Wei B.-R., Simpson R.M. (2014). Digital pathology and image analysis augment biospecimen annotation and biobank quality assurance harmonization. Clin. Biochem..

